# Advanced Sensors and Systems Technologies for Indoor Positioning

**DOI:** 10.3390/s22103605

**Published:** 2022-05-10

**Authors:** Riccardo Carotenuto, Demetrio Iero, Massimo Merenda

**Affiliations:** 1 Department of Information Engineering Infrastructures and Sustainable Energy (DIIES), “Mediterranea” University, 89122 Reggio Calabria, Italy; demetrio.iero@unirc.it; 2 Center of Digital Safety & Security, AIT Austrian Institute of Technology GmbH, 1210 Vienna, Austria; massimo.merenda@ait.ac.at

## 1. Introduction

There is an increasing interest about indoor positioning, which is an emerging technology with a wide range of applications. Accurate and real-time positioning enables augmented and mixed reality applications, human–machine and home automation gestural interfaces, and navigation in shopping centers. Relevant applications include robotics, acquiring the position of flexible arms, navigation of unmanned automatic vehicles, security, virtual fencing of sensitive locations, safety, and preventing accidents through the recognition of dangerous postures and positions in workers. Further fields of application include medicine, such as monitoring elderly people’s movements or rehabilitative exercises; logistics, such as the positioning of goods in warehouses; sport, such as monitoring body and limb position during training exercises and in game consoles.

Currently, research effort needs to be directed to new algorithms, architectures, sensor technologies, coverage, power consumption, size, and increased spatial and temporal resolution of indoor positioning systems based on the physical and economic constraints of various applications. In this framework, we are glad to edit this Special Issue on “Sensors and Systems for Indoor Positioning”. Original contributions focused on systems and technologies to enable the indoor applications listed above are welcome.

There are many challenges in this area that need to be solved or improved. Research effort needs to be directed to new algorithms, architectures, sensor technologies, coverage, power consumption, size, and increased spatial and temporal resolution of indoor positioning systems, based on the physical and economic constraints of the various applications. In this outline, the Special Issue on “Sensors and Systems for Indoor Positioning” of the *Sensors* journal seeks to explore original contribution on systems and technologies to enable the indoor applications listed above are welcome.

From several received manuscripts, eleven original and high-quality papers were selected to be included in this Special Issue, each one reviewed by multiple expert reviewers and passed through several rounds of peer review.

## 2. Relevant Contributions

In [1], a carrier phase technology in wireless orthogonal frequency division multiplex (OFDM) systems is applied to improve ranging and positioning accuracy. Carrier phase measurement is a ranging technique that uses the phase difference between the received signal and the transmitted signal. Compared with positioning systems using only time of arrival (TOA), carrier phase information has a higher resolution and is more accurate, providing indoor high-precision positioning. Carrier phase ranging is widely used in global navigation satellite systems (GNSS) systems but is not yet commonly used in OFDM systems. Applying this technology can significantly improve positioning accuracy. However, using the OFDM carrier phase has two problems that the authors intend to solve: (1) phase measurements in multipath environments and non-line-of-sight (NLOS) propagation; (2) integer ambiguity resolution in real-time positioning applications.

The paper presents a ranging scheme based on the carrier phase in a multipath environment; it analyzes the effect of multipath propagation on phase measurement and reports a correlation profile-based carrier phase measurement method. An extended Kalman filter (EKF) algorithm is also presented to estimate the integer ambiguity by SD carrier and TDOA measurements. The algorithm also considers the effect of NLOS error and mitigation efforts. Simulation shows that the proposed algorithm quickly solves the integer ambiguity even when NLOS errors occur. The carrier phase measurements combined with the accurately valued integer ambiguity led to a positioning error below 30 cm for 90% of the terminals.

The effective implementation of a UHF-RFID Smart Gate, an identification point placed at warehouse key points for forklift monitoring, is presented in [2]. The system is part of the I-READ 4.0 project aimed at developing an integrated and autonomous Cyber-Physical System for the automatic management of large warehouses with a high-stock rotation. The management of assets and forklifts is possible thanks to a network of Radio Frequency Identification (RFID) readers operating in the Ultra-High-Frequency (UHF) band.

The UHF-RFID Smart Gate consists of a checkpoint infrastructure based on RFID technology to identify forklifts and their direction of transit. The authors present an implementation with a single reader antenna, thus reducing infrastructure complexity and cost. The action classification method exploits the signal phase backscattered by RFID tags placed on forklifts, allowing the classification of two movements (entering or leaving a certain area) that the forklift can perform with respect to the gate.

The proposed system does not require calibration procedures, it does not need long computational times, and it can be implemented with commercial-off-the-shelf (COTS) components. The performance and the method capabilities were demonstrated in a real warehouse, and in 100% of cases, the forklift was correctly detected, and a 98% classification accuracy was achieved when the forklift speed ranged between 0.5 m/s and 1.5 m/s. 

In [3], a new technique was presented to measure the distance between an emitter and a receiver, which is based on the different attenuation levels that ultrasonic signals of different frequencies undergo when propagating in the air.

Distance measurements are usually performed by measuring the Time of Flight (TOF) of an ultrasonic signal traveling from an emitter to receiving sensors. However, this requires close synchronization between the emitter and the sensors. This synchronization is usually conducted using a radio or optical channel, which requires additional hardware complexity, while for many applications, low-cost small lightweight sensors are required.

Intending to reduce the complexity of the measurement process and of the sensors, the paper proposes an innovative technique that measures the distance between emitter and receiver from the amount of attenuation suffered by signals emitted at different frequencies, without the need for any synchronization between them.

Simulation results showed that, using a 0.5 mm diameter emitter aperture, a ranging error of less than 2.75 cm and a mean error of 1.25 cm can be achieved. The technique does not reach the level of accuracy of other techniques but works in the absence of synchronization without limits on the distance measurement rate, with an unlimited number of sensors using the same emitter and with reduced computational power and device dimensions.

In [4], the authors compare two methods for the acoustic indoor localization of persons based on the time difference of arrival of the first-order reflection to interpret the returned signals in a small office room. They draw the approach from bats which can perceive the incoming reflected wave’s direction. The first method is Direct Intersection, which determines a coordinate point based on the intersection of spheroids defined by observed distances of high-intensity reverberations. The second method, Sonogram analysis, overlays all channels’ room impulse responses to generate an intensity map for the observed environment.

The authors investigate the two algorithms and both approaches yield mean distance localization errors ranging between 0.3 and 0.9 m. Direct Intersection shows a higher precision, while the Sonogram Estimation method provides more accurate results. Moreover, the former method has a lower computational cost and performs faster with comparable precision and accuracy.

In [5], a deep learning solution involving a clustering processing scheme in a fingerprint indoor positioning system was developed. Wi-Fi fingerprint-based positioning systems have a simple layout and a low cost; however, the multipath propagation of signals caused by obstacles, interference of moving objects, and changes in Wi-Fi APs affect the positioning accuracy based on a received signal strength indicator (RSSI) with traditional dataset and a deep learning classifier. To overcome this issue, the authors propose a clustering-based noise elimination scheme (CNES) for RSSI-based datasets, in which the dataset is preprocessed and noise samples are removed.

Experiments carried out in a dynamic environment showed that applying CNES to the test database will increase the average positioning accuracy up to 22.4%, archiving a positioning accuracy of 90.4%, which is much higher than the accuracy of the dataset without pre-processing.

A smartphone-based navigation and information service for a University library employing Wi-Fi fingerprinting is developed in [6]. The motivation of this study is to help students, employees, and visitors of the TU Wien University to find the correct bookshelf. The authors carried out a study of the availability, performance, and usability of Wi-Fi in areas of the library using different smartphones in different modes, such as static, kinematic, and stop-and-go, evaluating positioning accuracies in the various modes. The investigations showed that Wi-Fi fingerprinting can be used to achieve positioning accuracies on the meter level. Accuracy can be increased by the installation of additional access points to provide better distribution and geometry for localization and also by deploying additional hardware based on low-cost Raspberry Pi units that broadcast and receive Wi-Fi signals. 

In [7], a three-dimensional visible light positioning system with multiple photodiodes and reinforcement learning (RL) is demonstrated. The system can realize accurate 3D positioning without the need of data for offline training. The authors propose and compare experimentally three methods developed to improve the 3D positioning accuracy over a basic 3D positioning model based on the RSSI trilateration without RL.

The experimental results show that the three RL-based methods outperform the basic one, providing higher position accuracy. Among the three methods, the third, which is a combination of the first two, offers the highest positioning accuracy, with an average positioning error of 2.6 cm and at least 20% improvement compared to the basic model.

In [8], the authors propose an indoor localization system based on an infrared angle-of-arrival (AoA) sensor network for accurate and inexpensive real-time. The authors attempt to overcome the disadvantage of state-of-the-art indoor localization systems relying on complex NLOS signal propagation with multiangulation and multilateration methods that have high installation costs, computational demands, and energy requirements. The paper presents a novel sensor utilizing infrared (IR) signal in the line-of-sight (LOS) context using the AoA technique that avoids NLOS propagation issues by exploiting the concept of the wireless sensor network (WSN). 

To demonstrate the proposal, a supermarket cart navigation system was realized as a proof-of-concept using an IR-AoA sensor prototype, server-side component, and an application for smartphones and smartwatches. The localization performance ranged from centimeter-level accuracy achieved in a static context to 1 m mean error in a mobile cart context. The implementation demonstrated that inexpensive and easily deployable wireless sensors nodes can be utilized to provide appropriate localization accuracy.

In [9] an adaptive residual weighted K-Nearest neighbor (WKNN) fingerprint positioning algorithm based on visible light is proposed. The WKNN algorithm is a commonly used fingerprint positioning algorithm for which its difficulty consists in the optimization of K to obtain the minimum positioning error. The authors propose an adaptive algorithm in which, initially, the target matches the fingerprints according to the RSSI, and K is a dynamic value according to the matched RSSI residual.

Simulation results show that the proposed algorithm presents a reduced average positioning error when compared with random forest (81.82%), extreme learning machine (83.93%), artificial neural network (86.06%), grid-independent least square (60.15%), self-adaptive WKNN (43.84%), WKNN (47.81%), and KNN (73.36%). Moreover, it achieves a significant reduction in positioning error while maintaining lower algorithm complexity.

In [10], the use of software Field II is proposed to simulate signal aberration and ranging error in ultrasonic indoor positioning applications. Ultrasonic systems have already been demonstrating their effectiveness in achieving high positioning accuracy and refresh rates, but attention must be paid to certain aspects of signal propagation. In this paper, Field II, an acoustical simulation software that is well-established in medical imaging, has been applied to the acoustic field in the air for the evaluation of ranging techniques.

In this study, it is shown how a typical chirp signal used in ultrasonic positioning systems undergoes a shape aberration depending on the shape and size of the transducer and on the angle under which the transducer is seen by the receiver. Such signal shape aberrations produce results affected by a much greater error than expected. The spatial distributions of the ranging error are provided, showing favorable low error regions. The work also demonstrates that particular attention must be paid to the design of the acoustic section of the ultrasonic positioning systems, considering both the shape and size of the ultrasonic emitters and the shape of the acoustic signal used.

In perspective, the advantages of the proposed approach are the possibility of examinations, while in the design phase, advantages include the acoustic field over time in the region of interest as a function of the aperture and the type of signal emitted and the capability to easily test several algorithms in different operating situations.

In [11], a study on a recursive algorithm for indoor positioning using pulse-echo ultrasonic signals was investigated. Ultrasounds are widely used for real-time applications in short-range communication systems and one of the parameters widely used is TOF, which can be evaluated by using different techniques. In the paper, a nonstandard cross-correlation method is investigated for TOF estimation, with a procedure based on the use of template signals to improve the accuracy of recursive TOF evaluations.

Experimental results were compared with both the standard threshold and cross-correlation techniques, showing an average improvement of 30% and 19% in terms of standard error, and an enhancement in repeatability of about 10%. However, an increase of 70% in computational load has been estimated in the evaluation of TOF.
